# Associations between Physical Activity and Academic Competence: A Cross-Sectional Study among Slovenian Primary School Students

**DOI:** 10.3390/ijerph19020623

**Published:** 2022-01-06

**Authors:** Joca Zurc, Jurij Planinšec

**Affiliations:** 1 Department of Pedagogy, Faculty of Arts, University of Maribor, 2000 Maribor, Slovenia; 2 Department of Elementary Education, Faculty of Education, University of Maribor, 2000 Maribor, Slovenia; jurij.planinsec@um.si

**Keywords:** physical activity, leisure time, sports, academic competence, late childhood, multiple regression analysis

## Abstract

Physical activity has beneficial effects on overall academic performance in children. However, there is a lack of evidence regarding how the individual characteristics of physical activity interact with other confounding variables of academic competence. Leisure-time physical activity with potential confounders—such as developmental, behavioral, family, and school factors, predicting overall, mathematical, and reading academic competence—was studied in a random sample of 1520 Slovenian primary school students in grades 4–6 (51.9% female; mean age = 10.4 years; SD = 0.93). A structured self-reported questionnaire was used to gather data on the children’s leisure-time physical activity and social-demographic variables, while academic competence was measured by teachers using the SSRS Academic Competence Evaluation Scale. The findings showed that children engage in physical activity most days a week, with moderate-intensity and unorganized activities. It was predicted that engaging in physical activity would lead to an increase in academic performance by 4.2% in males (*p* = 0.002) and 3.2% in females (*p* = 0.024), but after fully adjusting the model for controlling confounding variables, the prediction increased to 81.1% in females and 84.1% in males (*p* < 0.001). The frequency and intensity of physical activity, the absence of digital games, and attending sports clubs seem to have the most beneficial effects in terms of academic competence in school children, among other relevant confounders mediating in this complex relationship.

## 1. Introduction

Physical activity (PA) is a crucial factor in children’s development and health. Performing 60 min of regular moderate-to-vigorous PA ever day throughout the week, including vigorous aerobic activity and strength exercises for muscles and bones at least 3 days per week, improves physical fitness, cardiometabolic health, bone health, and mental health in children [1,2]. In addition, PA has a long-term positive impact on cognitive functioning and academic performance, which is particularly evident in children [3,4,5,6,7,8,9,10,11,12,13,14,15,16]. When children spend more time on physical activities, it can lead to higher grade point averages and more efficient curriculum implementation, even if the time spent on other school subjects or the free time spent on learning academic material is reduced [6,9,17]. Positive associations were found in various academic competencies, such as overall or total academic achievement (i.e., overall average achievement across various school subjects over a certain period based on teacher evaluation or standardized tests), mathematical achievement, and reading achievement [5].

Over the past 20 years, many researchers have attempted to identify the mechanisms that explain the impact of PA on academic performance. PA is a potent stimulator of molecular and cellular components that underlie brain structure and function and, in particular, are responsible for complex cognitive processes [4,18]. A systematic review by Tomporowski et al. [5] showed that PA enhances children’s executive functions (i.e., processes required to select, organize, and properly initiate goal-directed actions) and, therefore, provides a simple but important mechanism for promoting those aspects of children’s cognitive development that are important for both academic achievement and problem solving later in life. For example, more physically fit children showed a greater ability to upregulate neural processes that support executive control to meet task demands and maintain performance [19]. PA positively affected attention and concentration [20], working memory, cognitive processing speed [21], psychomotor skills [17], self-awareness [22], metacognitive processes [23], and adaptive classroom behavior [24]. The evidence suggests that both quantitative (e.g., intensity, frequency, and duration) and qualitative (e.g., problem-solving complex demands) types of PA enhance cognitive processing [23].

However, not all studies have reported positive associations between PA and academic performance in primary school children. For example, in a systematic review by Singh et al. [25], 40% of the intervention studies reported little or no positive impact of PA on children’s academic performance. Similarly, in a systematic review by Rasberry et al. [10], only half of the studies found a significant positive association, 48% found no significant correlations, and 1.5% of the studies even showed a significant negative correlation between PA and academic performance. Positive associations between PA and academic performance were reported more frequently when the academic performance assessed by teachers or children’s grades were used as an outcome measure [5]. In addition, cross-sectional and longitudinal studies showed more favorable results, while results from controlled experiments were mixed [18]. Finally, the effect size of significant associations between PA and academic performance was generally found to have minor to moderate effects on academic performance [15]. Lima et al. [26] reported that physical activity intervention was effective only in students who had failed a school year. These findings suggest that other confounding factors, such as developmental [5,15,27], behavioral [24,28], and contextual factors, in the family environment [15,25] and the school environment [26,29], may have a substantial impact on academic performance through interaction with PA. However, which confounding factors might play an important role and how they interact with PA and influence school children’s academic performance are still the focus of debates.

Current evidence on the relationship between PA and academic performance appears inconclusive and limited. Instead of a growing number of systematic reviews, findings are rarely based on high-quality studies with representative samples, using standardized assessment tools of a valid and reliable measure of PA and academic performance. Moreover, they often do not take into account confounding factors or relevant moderators for academic performance. As a result, many aspects of this relationship remain unclear, including the characteristics of PA (e.g., frequency, intensity, duration, and type) that most favorably affect academic achievement [5,18,19,25]. In addition, better insight into the effects of sedentary behavior and screen time would greatly expand the understanding of the links between PA and academic performance in school children [15,25]. Given the need to clarify the nature of the associations between PA and children’s academic performance, we aimed to determine in this study: (1) how leisure-time PA interacts with other potential confounding variables, such as developmental, family, and school contextual factors; (2) which individual characteristics of leisure-time PA play a critical role in predicting children’s academic performance; and (3) whether there are any differences in the relationships between different academic competencies, such as overall academic competence, mathematical competence, and reading competence, and between females and males.

Using a cross-sectional survey on a representative sample of Slovenian primary school children in grades 4–6 with standardized measured academic competencies and multiple regression analysis, we identified the characteristics of leisure-time PA that may predict academic outcomes in primary school children.

## 2. Materials and Methods

This is a non-experimental study with a cross-sectional design.

### 2.1. Participants

A total of 16 primary schools from the population of all primary schools in Slovenia (*n* = 454) were included. We used multistage sampling, i.e., a three-stage random sampling procedure. First, primary schools from all 12 statistical regions of Slovenia were randomly selected according to the size of the region: 3 primary schools from the Central Slovenia region; 2 schools each from the Drava and Savinja regions; and 1 school each from the Mura region, the Carinthia region, the Middle Sava region, the Lower Sava region, the Southeast Slovenia region, the Upper Carniola region, the Inner Carniola-Karst region, the Coastal Karst region, and the Gorizia region. In the second phase, we selected 4th- to 6th-grade classes from the randomly selected schools. These were primary schools where permission to interview the children was obtained from the principal and class teachers of the respective classes. The third stage of sampling included all the children from the selected classes whose parents had signed a written consent to participate in the study and who were present in the school on the day the study was conducted. In total, 2192 Slovenian primary school students in grades 4–6 were invited to participate in the study. Among these, 1782 children participated in the study, but 262 participants were excluded from the analysis due to incomplete responses or other missing data in the assessment. Thus, the data of 1520 children and their class teachers were used in the final analysis.

The results of our study can be generalized to the population of children in grades 4–6 of the second cycle of education in primary schools in Slovenia.

### 2.2. Study Procedures

After prior consultation with the school administration, data were collected from school children in randomly selected primary schools during regular school hours. A questionnaire on leisure-time PA was distributed to the children whose parents had provided written consent to participate in the study and who were present in the school on the day of data collection. Participants completed the questionnaire independently and individually. The PA questionnaires were distributed to the children, who had 30 min to complete and return the questionnaires.

The academic competence questionnaire was distributed to all class teachers in the participating classes. The teachers were asked to complete the questionnaire for each child in their class whose parents had signed a written informed consent form to participate in the study and had filled in the questionnaire on leisure-time PA. The class teachers completed the questionnaires independently and individually in the school (in their offices or unoccupied classrooms) when they could do so without interruptions. The time for completing the questionnaires was not limited but varied according to the number of participating children in each class. All teachers voluntarily participated in this study, receiving no funding or other compensation for their participation.

The first author, who has expertise in survey methodology and primary education (grades 1–6), conducted all the measurements. She was present during the completion of the questionnaire to help both children and teachers with possible questions.

### 2.3. Measures

#### 2.3.1. Physical Activity Questionnaire

Based on previous studies [13,30,31,32,33,34], a structured, closed-ended questionnaire was designed and used to collect data on the PA of participating children during leisure time. The following dependent variables were examined: frequency of leisure-time PA per week, intensity of PA, frequency of unorganized PA (performed alone or with friends or with family), frequency of organized PA (extracurricular sports activity and/or activity in a sports club), and frequency of PA by popularity (football/soccer, walking, cycling, and jogging). Following the recommendations of previous studies on the importance of sedentary behavior in understanding the context and complexity of academic achievement [15,25], three sedentary variables assessing screen time were included in our measurements: daily watching of television, daily computer use, and daily playing of digital games. The dependent variables were measured on assessment interval scales, with 1 indicating negligible presence or the absence of a criterion and the highest value indicating the maximum presence of that criterion.

The validity of the leisure-time PA questionnaire was tested in a previous study [35]. Principal component analysis showed three-dimensional construct validity of children’s self-reported PA, consisting of (1) physical inactivity (sedentary behavior), (2) parental PA level, and (3) children’s PA level. All three components explained a total of 68.8% of the variance in children’s leisure-time PA. The first principal component explained 27.7% of the variance and was indicated by sedentary variables, such as daily computer use (0.67), daily playing of digital games (0.64), and daily watching of television (0.60). The second principal component explained 25.4% of the variance and was indicated by the mother’s (0.59) and father’s (0.56) PA. Finally, the third principal component explained 15.7% of the variance and consisted of the intensity (0.58) and frequency (0.54) of the children’s PA.

#### 2.3.2. Academic Competence Assessment Tool

The children’s academic competence was assessed using Gresham and Elliott’s Social Skills Rating System (SSRS) [36]. The SSRS is a valid and reliable measurement tool for assessing children’s social skills and has emerged from longitudinal research on children’s social behavior and the expansion of an instrument developed in 1984, the Teacher Ratings of Social Skills (TROSS). The SSRS was tested in 1987 and underwent national standardization in 1988 involving 4170 children, 1027 parents, and 259 teachers. The items of the SSRS are based on extensive empirical evidence from child development, clinical psychology, educational psychology, and special education concerning students with disabilities [36].

This standardized test consists of three assessment forms—for the student, parent, and teacher—covering three different scales: the Social Skills Scale (student self-report, parent form, and teacher form), the Problem Behavior Scale (parent form and teacher form), and the Academic Competence Scale (teacher form). Only the SSRS teacher form was used to assess the academic competence of school children in this study. The scale included nine dependent variables that reflected each student’s academic functioning from the teacher’s perspective: overall academic performance, reading performance, mathematical performance, reading skills, mathematical skills, child’s motivational level, parental support, overall cognitive functioning, and overall classroom behavior.

All variables were scored on a 5-point scale to rank the child’s academic performance compared to the other students in the class. The number 1 indicates the lowest 10% of students in the class, the number 2 the next-lowest 20%, the number 3 the middle 40%, the number 4 the next-highest 20%, and the number 5 the highest 10%. The highest possible score was 45 points, and the lowest possible score was 9 points.

Based on the SSRS test, children were placed into one of three academic achievement groups corresponding to developmental norms: below average (9–15 points), average (26–44 points), and above average (45 points). Gresham and Elliott [36] developed the norms based on the frequency distribution of raw scores from the SSRS standardization sample and stratified them by age, gender, and disability status of the children. In the process, the plotted curves were adjusted to remove any possible bias due to sampling and measurement error and the standard scores with a mean of 100 and a standard deviation of 15 were assigned to specific percentile ranks.

The reliability of the SSRS was estimated using internal consistency and test–retest [36]. First, high internal consistency or homogeneity of the items of the Academic Competence Scale was demonstrated with Cronbach’s α value of 0.95 for females and males. Second, test–retest reliability was measured by assessing the standardization sample of the same group of participants 4 weeks after the initial standardization assessments. For the teachers’ assessment of the students’ academic competence, significant evidence of stability and consistency over time was found with a test–retest correlation of 0.93.

Several validation strategies were used to develop the SSRS [36]. First, content validity was established using the principles of a comprehensive literature review and a panel of experts [37]. A three-stage process for assessing content validity of the Academic Competence Scale was applied: (1) developmental stage (an experienced researcher nominated a pool of items based on the literature review); (2) judgment and quantifying stage (teachers rated the importance of each item on the scale using the categories of *not important*, *important*, and *critical* in terms of behavior for a child’s school success); and (3) revising and reconstructing stage (the final instrument was developed). This rigorous assessment process helped evaluate the selected items and their rating based on the degree to which they are relevant and representative for the target construct of academic competence assessment [36,37]. Second, criterion-related validity was tested using three similar social behavior assessment scales [38,39,40]. Correlations between these scales and the Academic Competence Scale ranged from 0.26 to 0.72, with an average correlation of 0.56. The moderate-to-high correlations indicate that they measure similar constructs and support criterion-related validity for the SSRS teacher form of assessment academic competence in elementary-school-age children. Finally, construct validity was determined using factor analysis (principal component method, Kaiser-Guttman’s criterion, and direct oblimin rotation) on primary school children from the standardization sample to determine the underlying dimensions of the Academic Competence Scale. The nine items on the scale measuring academic achievement were captured in only one factor, labeled *academic competence*. The items on the scale had high factor loadings, ranging from 0.62 to 0.93, with the highest loadings found for overall academic performance (0.93), intellectual functioning (0.91), reading competence and skills (0.90), and mathematical competence and skills (0.90). Although each of these items was highly correlated with the others, the authors decided to keep all items on the scale because each item seemed to have social validity [36]. Related studies [41,42,43] have shown the SSRS test’s comparable validity, reliability, and objectivity.

#### 2.3.3. Controlling Variables

Controlling for potential confounding variables related to developmental, family, and school characteristics of participants included gathering data reported by children (e.g., gender, age, grade, and type of school), their teachers (e.g., the child’s developmental disability, educational level, and working years in school), and their parents (e.g., maternal and paternal educational levels and the number of children in the family). In addition, parental encouragement and students’ motivation to succeed academically as confounding variables were extracted from the academic competence assessment tool, evaluated by the teachers.

#### 2.3.4. Pilot Study

A pilot study tested the PA questionnaire and the SSRS test for validity, reliability, objectivity, and sensitivity. Both measurement instruments were improved and standardized based on the pilot study results for Slovenian primary school children in grades 4–6. In the pilot study, 151 students from one of the selected schools in the Upper Carniola region participated. A high level of internal consistency was calculated for both assessment systems: Cronbach’s α values for children’s academic competence and leisure-time PA were 0.965 and 0.841, respectively.

In the pilot study, the SSRS was translated into Slovenian. The first draft was prepared by the first author of this article. Participants in the pilot study were explicitly asked to evaluate the items of the SSRS in terms of clarity, comprehensibility, and cultural sensitivity. Based on their feedback, some linguistic revisions were made to the Academic Competence Scale to improve the transparency of the Slovenian version.

The pilot study showed a selection of relevant items in both measurement instruments to determine the dimensions of children’s PA behavior and academic competence.

### 2.4. Considerations of Ethical Principles

This study is based on the ethical principles for research involving human subjects, as set out in the Declaration of Helsinki. Personal data were collected and protected under the provisions of the Personal Data Protection Act [44]. The ethical aspects of the study were assessed by the Scientific Council of the Slovenian Research Agency, with the involvement of Slovenian and foreign peers in the evaluation of the submitted project. The study was rated as ethically acceptable. Considering the absence of sensitive ethical issues, the voluntary and anonymous nature of the survey, and the assessment by the Scientific Council of the Slovenian Research Agency, assessment by an ethics committee was not required. However, the parents of all participating children provided written informed consent for their child’s participation in the study prior to data collection. All participating teachers also provided written informed consent to participate in the study. The only person who had access to the participants’ answers was the first author of this article, who maintained and protected the anonymity of the participants. All research presented respects the confidentiality and anonymity of the participants.

### 2.5. Data Analysis

Data on children’s leisure-time PA and academic competence were analyzed using IBM SPSS version 28.0 (IBM, Armonk, NY, USA), including descriptive statistics (frequency, percentage, mean, and standard deviation), bivariate statistics using the t-test for independent samples, Pearson’s chi-square test and one-way analysis of variance, and multiple regression analysis. The level of statistical significance was set at *p* ≤ 0.05.

The *t*-test for independent samples and Pearson’s chi-square test were performed to detect possible differences in the levels of PA and academic competence between the sexes. Since our bivariate analysis showed that girls and boys differ in terms of leisure-time PA and academic competence, a multiple regression analysis was conducted for girls and boys separately. One-way ANOVA was used to first compare the three groups of students with different levels of academic competence (below average, average, and above average). The three levels were calculated based on the total score of the Academic Competence Scale of the SSRS test (teacher form) according to Gresham and Elliott [36]. Based on the results, only variables of leisure-time PA with statistically significant associations with academic competence were selected for modeling in the multiple regression analysis. Since the Pearson correlation between the selected variables of PA level and academic competence were statistically significantly linear in most cases, their relationships were examined in more detail using linear regression analysis.

Multiple regression analysis aimed to find a model of the characteristics of leisure-time PA and the potential confounding variables that best predict the academic competence of primary school children in grades 4–6. Five models were applied using Enter multiple regression analysis that included leisure-time PA and the potential confounding variables to predict academic competence (Table 1). All series of regression models were run separately for females and males and three types of academic competence: general academic, mathematical, and reading. Mathematical competence and reading competence were calculated as the mean academic performance score and ability score in each subject.

## 3. Results

The survey results of Slovenian primary school children in grades 4–6 are presented in three sections: (1) characteristics of the study participants, (2) academic competence of the participants, and (3) associations between PA and academic competence in the studied group.

### 3.1. Characteristics of Study Participants

Of the 2192 Slovenian primary school students in grades 4–6 who were invited to participate in the survey, 69.3% (*n* = 1520, 51.9% female) provided complete data for the analyses. The academic competence of eligible participants was assessed by their class teachers (*n* = 123, 88.6% female). Table 2 shows the characteristics of the participants included in the data analysis. On the teacher form of the SSRS test, 8.5% of the participants were identified as having a developmental handicap. The predominant handicaps were learning disabilities and behavioral problems. More handicaps were found in boys (12.8%).

The results of the participants’ leisure-time PA are presented in Table 3. Slovenian primary school children in grades 4–6 are physically active most days of the week (4.93 ± 1.23), with moderate-intensity and unorganized activities with friends (2.44 ± 0.61) or activity performed alone (2.36 ± 0.61). When asked about the popularity of 18 sports, children ranked football first (2.12 ± 3.14), followed by three outdoor activities: walking, cycling, and running. Watching television was the most common sedentary behavior. Statistically, boys were significantly more likely to report being more intensely physically active and more frequently engaged in sports clubs compared to girls.

Cycling and family-based physical activities were more common among girls than boys, while all three sedentary behaviors were significantly more prevalent among boys compared to girls (*p* < 0.001) (Table 3).

### 3.2. Academic Competence of Participants

Students’ academic competence was measured by their teachers using nine items on a scale of 1 to 5 (Table 4). On average, all variables were rated highly by the teachers. Parental encouragement (4.06 ± 0.99), general classroom behavior (3.92 ± 1.04), and student motivation to succeed academically (3.89 ± 1.06) were the most recognized characteristics of Slovenian school children’s academic performance. The lowest scores were found for mathematical achievement (3.75 ± 1.10) and mathematical ability (3.78 ± 1.08). In all individual variables examined and in overall academic competence, statistically, girls achieved significantly higher performance levels than boys. The gender differences were significant in all academic competencies except mathematical ability.

Based on the total score of academic competence, the students were divided into three groups using the standard scores and percentile ranks determined by Gresham and Elliott [36]. Most of them scored average values in academic competence (*n* = 1065, 68.8%). A chi-square test confirmed a statistically significant higher level of academic competence in girls compared to boys (*p* < 0.001) (Table 5).

### 3.3. Associations between PA and Academic Competence

The gender differences in leisure-time PA (Table 3) and academic competence (Table 4) presented above provided the impetus for a separate analysis of the relationships between PA and academic competence for girls and boys. The relationship between the phenomena under investigation was first tested using one-way ANOVA and various multiple regression models later.

Table 6 shows the results of the one-way ANOVA for the relationship between students’ PA level and academic competence. The statistically significant differences between groups of students with different levels of academic competence were found for frequency of daily computer use, daily playing of digital games, frequency and intensity of leisure-time PA, frequency of walking, participation in organized sports activities, and PA performed alone. For all significant associations, the highest levels of PA and the lowest levels of sedentary behavior were found in students with above-average academic competence. The only exception was the frequency of walking, which increased with decreasing academic competence.

The results of one-way ANOVA were used as a criterion for selecting leisure-time PA variables for multiple regression analysis. Only variables that showed statistically significant associations with academic competence were selected for modeling using multiple regression analysis (Table 7).

Three criterion variables, defining overall academic competence, mathematical competence, and reading competence, were used in five different multiple regression models for girls and boys. The models first included the variables for leisure-time PA and then gradually added potential confounding variables as predictor variables for academic competence, as shown in Table 7.

All models for boys and most models for girls showed statistically significant associations between leisure-time PA and academic competence. *R*^2^ showed the strongest prediction for overall academic competence for girls (3.2–81.1%) and boys (4.2–84.1%). The highest explanatory prediction for overall academic competence was found in model 5 (81.1% for girls and 84.1% for boys), which included the variables for leisure-time PA and all confounding variables. In contrast, model 1, which included only leisure-time PA variables, explained ~3% of the variance in overall academic competence for girls and ~4% for boys. The incremental addition of confounding variables to the models increased their prediction and explanation of factors of academic competence. The lowest prediction was found for mathematical competence. Model 3 explained 43.9% of the mathematical competence, which was the highest prediction for this competence in girls. The models for boys showed stronger predictions than the models for girls.

Since leisure-time PA variables for predicting academic competence in primary school were the focus of our study, we reported the association between the variables measuring the PA characteristics and overall academic competence in model 1 (Table 8). The frequency of PA among girls (*p* = 0.024) and participation in sports clubs among boys (*p* = 0.002) were statistically significant predictors of academic achievements among Slovenian 9- to 11-year-olds. The resulting *β*-value suggests that for every 1 SD increase in the frequency of leisure-time PA, girls’ overall academic competence increases by 0.10 SD.

That leisure-time PA significantly predicts academic competencies was also found in other models. For girls, spending more time on PA predicted higher overall academic competence after adjusting for confounding factors in model 2 (0.14 SD increase) and model 3 (0.11 SD increase). Similarly, girls’ mathematical competence could be predicted by the frequency of PA (0.10 SD increase) after adjusting for confounding factors in model 2 (0.16 SD increase) and model 3 (0.12 SD increase). Playing digital games was associated with lower reading competence in girls in model 1 (−0.12 SD decrease) and model 2 (−0.09 SD decrease). In contrast, increased frequency of PA, adjusted for confounding variables, predicted an increase in girls’ reading literacy in model 3 (0.08 SD increase) and model 2 (0.11 SD increase).

For boys, the overall academic competence could be predicted by the frequency of PA performed alone (0.10 SD increase) and the frequency of activity in a sports club (0.11 SD increase) in model 2 and the intensity of PA in model 4 (0.55 SD increase). Similar results were found for both mathematical competence and reading competence. For mathematical competence, an increase in the frequency of PA in a sports club (model 1 = 0.14 SD increase; model 2 = 0.11 SD increase) and PA performed alone (model 2 = 0.13 SD increase) and a decrease in the intensity of PA (model 2 = −0.10 SD decrease) predicted better performance in mathematics when the overall PA was controlled for the confounding variables of grade level and developmental disability. In contrast, the intensity of daily PA predicted a 0.78 SD increase in boys’ reading competence in fully adjusted model 5 and a 0.83 SD increase in adjusted model 4. In addition, boys’ reading competence could be predicted by the frequency of PA performed alone (0.09 SD increase) in model 2 and membership in a sports club (0.12 SD increase) in model 1.

## 4. Discussion

Conducting multiple regression in this study revealed significant associations between leisure-time PA and higher overall academic competence, mathematical literacy, and reading literacy among female and male primary school students in grades 4–6. Students with above-average academic ability meet or exceed some of the WHO recommendations [1] for PA. Compared to the average and below-average groups, students with above-average academic ability report a significantly higher frequency and intensity of leisure-time PA, a higher preference for walking and participation in sports clubs, and less sedentary behavior in front of screens.

Although it is statistically significant, leisure-time PA itself explains only 3% of academic competence in girls and 4% in boys. The prediction becomes much clearer when the model is adjusted for confounding variables, such as developmental, family, and school factors. When confounding factors are added, the model can explain more than 80% of the variance in academic competence. In the following sections, we discuss which confounding variables have the greatest impact on the model and act as mediators between PA and academic competence and which elements or characteristics of PA are most strongly related to academic competence in school-age children. Finally, we present some strengths and limitations of the study.

### 4.1. Mediators Linking PA to Academic Competence

Our results suggest that children’s grade level and developmental handicap, along with the family size and parental encouragement for academic success, play a significant mediating role between PA and academic competence. This is true for all academic competencies and both males and females. Adding the confounding variables from the school environment into the model could reduce the prediction for girls’ mathematical and reading competencies. For boys, however, all models remained significant for all academic competencies, with the prediction increasing when other confounding variables were added, especially classroom behavior and motivation to succeed academically.

A positive impact of leisure-time PA on academic competence when controlling developmental, family, and school confounders is consistent with emerging evidence [15,27,29,45]. Similar to our findings, a longitudinal study by Kyan et al. [46] found a statistically significant positive correlation between PA and academic performance in boys but not to the same extent in girls. These findings suggest that in addition to PA, other predicted factors, such as family context [15,27], parental engagement [47], students’ academic expectations [48], coping with behavior problems [28], and enhancing the pedagogical skills of teachers [26], may contribute significantly to improving academic performance. Therefore, further studies should take a complex and holistic approach to considering children’s academic performance as an outcome of multiple factors and constructs.

### 4.2. Characteristics of Physical Activity as Predictors of Academic Competence

In this study, conducted on a representative sample of Slovenian primary school students in grades 4–6, the extent and intensity of leisure-time PA were reported according to the recommendations of WHO [1]. On average, the children were active 5 days per week, mostly with moderate-intensity and unstructured PA with friends or alone. Higher-frequency and higher-intensity PA and participation in organized physical activities were reported significantly more often by boys.

The partial coefficients of the multiple regression showed a significant, albeit small, effect size of the predictor variables for academic competence based on the total frequency of PA in leisure time for girls and involvement in sports clubs for boys. Schoolgirls who are more frequently physically active in their leisure time may benefit from better overall academic performance. Similarly, more frequent involvement in organized physical activities, such as sports clubs or sports associations, may lead to better academic performance for boys. Dapp and Roebers [22] came to a comparable conclusion that participation in structured or organized physical activities with sufficient frequency (at least 2 h per week) may be beneficial for the mathematical performance of school children in grade 4. In addition, longitudinal studies have found similar results for moderate-to-vigorous intensity PA, with higher-intensity PA being associated with better academic performance [5,15]. After controlling for confounding factors, some of the regression models in this study also indicated that the intensity of leisure-time PA is a significant predictor of academic competence, especially in boys. Khan and Hillman [19] explained this phenomenon from a neuroscience perspective by reporting that more physically fit children have a remarkable capacity for executive control, which supports the performance of goal-directed cognitive tasks underlying perception, memory, and behavior.

In addition, previous studies have shown a significant relationship between the type of PA and the academic performance of school children. For example, a comprehensive meta-analysis by Fedewa and Ahn [7] found that walking, among other aerobic physical activities, has the most significant positive associations with academic performance and cognitive outcomes. Similarly, a study of 2023 10-year-olds from north-western Slovenia showed that aerobic physical activities, such as hiking, swimming, and running, predominate among children who show above-average or average academic performance. In contrast, children with below-average academic performance are more likely to participate in ball sports, such as football and basketball [13]. In this study, no significant relationship was found between the type of leisure-time PA and academic competence. However, a group comparison between students with different academic competencies showed that the popularity of walking is higher in the above-average group than in the average and below-average groups.

Finally, notable implications were found for the relationship between academic competence and sedentary behavior. Although the time spent in front of the TV was the most common sedentary activity among the children in this study, it was not a significant predictor of academic competence. On the contrary, playing digital games on a daily basis led to a significant decline in academic performance, especially for girls in their competency in reading. The relationship between academic performance and sedentary behavior is not well established. Nevertheless, Jaruratanasirikul et al. [49] came to a similar conclusion that excessive digital game playing is positively correlated with below-average academic performance. It appears that leisure screen time may be an important factor in explaining lower reading skills and reading performance in primary school children. However, the relationship between sedentary behavior and academic performance should be closely and thoroughly investigated in future intervention studies.

### 4.3. Study Strengths and Limitations

The main strengths of this study were the large and representative sample with a high response rate of participants, which allowed generalization of the results to the entire population of Slovenian primary school students in grades 4–6; the objectively measured academic performance using a standardized assessment instrument; and a comprehensive set of confounding variables controlled for in the multiple regression models of the associations between leisure-time PA and academic competence. This combination of strengths contributes to the justification and credibility of the study’s findings.

However, this study also had a few limitations. First, the data collection method to assess children’s leisure-time PA was based on a self-report questionnaire. Data collection was controlled and guided by the principal researcher, with the support of class teachers in the field, to minimize bias due to misunderstanding or socially desirable responses from participants. However, Jurakić et al. [50] warn that although self-assessment is the most common method of data collection in cross-sectional PA studies, it can lead to overestimation of respondents’ fitness levels, especially among those with lower PA levels. Therefore, some studies [6,50] have suggested measuring PA in different domains, such as extracurricular sports activities, physical education classes, recess, physical activities in the classroom, physical activities related to transportation, and physical activities during leisure time. The fact that only one setting was observed for children’s PA in this study could be the reason that individual characteristics of PA can only explain academic competence to a limited extent.

Based on the frequency, intensity, time, and type of PA (FITT) principle [51], our study collected self-reported data on all four characteristics of children’s PA. However, the time or duration of PA was subsequently excluded from the analysis. The participants were asked to estimate how many hours or minutes per week they are active in sports and PA, including school and weekend days. The findings showed a lack of reliability of dispersion of the normal data distribution, suggesting that children may overestimate their time spent on PA. Hence, time as one of the main PA characteristics was not included in further analysis. Future systematic reviews and meta-analyses should investigate the correlation between these results and objectively measured intensity and duration of PA in children (e.g., using advanced technology), considering the different settings in which children’s PA may occur. In addition to the FITT principle, other characteristics of PA should be considered, such as organized vs. nonorganized forms of PA [24], the timing of PA during daily activities [52], and enjoyment in PA [53]. These could be additional invaluable factors for understanding the relationship between PA and academic performance in school-age children.

Second, the Academic Competence Scale was used to assess different aspects of children’s academic performance and was not limited to one school subject or the overall grade point average, as is common in previous similar studies [22,26,46]. The use of a standardized instrument to assess academic competence was a recognized strength of this study. However, the administration of this was quite demanding. Indeed, each of the participating teachers had to assess all the students in their class. This task took at least 1 h, in many cases with the support and encouragement of the researcher. One might suspect that the limited time and saturation of participants affected the biases in the study, as reflected in the overall high rating of the participants’ academic competence. In future studies, data collection should be carefully planned and specific training for teachers who will take on the role of assessors is strongly recommended. In studies on the relationship between PA and academic performance, the use of valid and reliable measures is strongly recommended [25]. The standardized Academic Competence Scale proved to be a relevant and methodologically sound instrument for assessing the academic performance of primary school children and is recommended for further use.

Third, previous studies [27,47] have shown that the parental education level is an influential factor in a child’s academic performance. In this study, data on the parental educational level were collected from the questionnaires filled out by the parents of the participating children. However, only a tiny sample of 48 families (3.2% of the total sample) provided information about the educational level of the mother or the father. Thus, since a high percentage of the data were missing, the parental education level was not applicable for further data analysis in our study. This limitation might have affected the results.

Finally, mathematical performance and mathematical skills were found to be the lowest among academic competencies for both genders. Moreover, regression models showed the lowest prediction of mathematical competence among girls. These findings contradict previous studies that demonstrate a significant relationship between PA and mathematical achievement. For example, in a systematic review of high-quality intervention studies, Singh et al. [25] found strong evidence of the positive effects of PA on mathematical performance. Substantial evidence indicates that PA improves the executive control of a task, which is particularly important for solving mathematical tasks [21,23]. The reasons for the inconsistency could be measurement error or the understanding of mathematical competence as a complex ability that is strongly influenced by other innate factors. Therefore, further studies are needed to investigate the relationship between PA and academic competence in different school subjects.

However, much remains to be learned about the effects of PA on academic performance. Therefore, qualitative and mixed-methods studies would also help understand how PA and academic performance interact with each other and other potential confounders. Different perspectives of children, their parents, and their teachers should be explored.

## 5. Conclusions

The findings of this study contribute toward elucidating the relationship between PA and academic performance by showing that sufficient frequency and intensity of leisure-time PA, together with participation in organized activities and limited time spent playing digital games, may significantly contribute to better academic performance in primary school children. However, although significant, the effect size of this relationship is modest when only PA characteristics are considered. The model becomes stronger when adjusted for potential confounding variables. Gender, grade level, developmental disability, family size, and parental encouragement for academic success appear to play the strongest mediating role between PA and academic competence. Therefore, these factors should be carefully considered when developing and implementing new school-based health promotion interventions. Multiple target groups should be addressed in interventions, including children’s families and the school environment, to ensure the sustained impact of PA on a child’s academic performance and overall well-being. It seems that interventions for girls could be effective when focusing on increasing the PA frequency and on decreasing sitting time when playing digital games, while interventions for boys are promising when encouraging their participation in sports clubs and engagement in vigorous PA.

The implications of the findings should be combined with established recommendations for PA for school-age children. Further high-quality intervention studies and systematic reviews are strongly recommended to clarify the association between PA and academic performance, considering several factors that mediate this complex relationship.

## Figures and Tables

**Table 1:** Five multiple regression models for predicting academic competencies.

	Model 1	Model 2	Model 3	Model 4	Model 5
Leisure-Time PA ^1^	X				
Developmental factors: School grade, developmental handicap	X	X			
Family factors: Parental encouragement, family size	X	X	X		
School factors: Type of school, level of teacher education and work experience	X	X	X	X	
Behavioral factors: Classroom behavior, motivation to succeed academically	X	X	X	X	X

^1^ Leisure-time PA characteristics were defined by frequency, organization form, intensity, and type of activities, selected as significant variables in a previous ANOVA.

**Table 2:** Characteristics of participants.

	Characteristics	Total Sample	Females	Males
		*n* (%)	*n* (%)	*n* (%)
Participants				
	Students	1520 (92.5)	789 (51.9)	731 (48.1)
	Teachers	123 (7.5)	109 (88.6)	14 (11.4)
Grade				
	4	580 (32.7)	290 (32.0)	279 (33.3)
	5	591 (33.3)	297 (32.8)	281 (33.5)
	6	604 (34.0)	319 (35.2)	278 (33.2)
Developmental handicap	Yes	109 (8.5)	28 (4.3)	78 (12.8)
	No	1178 (91.5)	624 (95.7)	531 (87.2)
Type of school ^1^				
	Central	1538 (86.6)	786 (86.8)	723 (86.3)
	Branch	238 (13.4)	120 (13.2)	115 (13.7)
Teacher’s level of education ^2^				
	Academy degree	55 (45.5)	23 (41.8)	30 (45.5)
	University degree	66 (54.5)	32 (58.2)	33 (50.8)
Family size (number of children)				
	1	146 (9.8)	82 (10.5)	63 (9.1)
	2	805 (54.2)	418 (53.5)	377 (54.7)
	3	399 (26.8)	212 (27.1)	184 (26.7)
	4	134 (9.0)	68 (8.7)	64 (9.3)
	≥5	3 (0.2)	2 (0.2)	1 (0.2)
		**Mean (SD)**	**Mean (SD)**	**Mean (SD)**
Participants’ age		10.40 (0.93)	10.40 (0.91)	10.41 (0.94)
Teacher’s work experience (years)		18.06 (10.74)	17.71 (10.76)	18.23 (10.66)

^1^ A branch school is usually a non-complete primary school attached to the central school. They have the same curriculum and administration, but a branch school is located in a different place, usually a smaller town. ^2^ The academy degree is based on the 2-year teacher training program after high school. This program has not been run since the early 1990s. The university degree is based on 4–5 years of higher education.

**Table 3:** Leisure-time PA of participants and differences between genders.

Leisure-Time PA	Total Sample (*n* = 1520)	Gender		
		Females (*n* = 789)	Males (*n* = 731)	*p*-Value ^6^
	Mean (SD)	Mean (SD)	Mean (SD)	
Frequency of leisure-time PA ^1^	4.93 (1.23)	4.85 (1.23)	5.01 (1.23)	0.009
Frequency of PA performed alone ^2^	2.36 (0.61)	2.38 (0.58)	2.35 (0.64)	0.275
Frequency of PA in the family ^2^	2.17 (0.63)	2.22 (0.64)	2.13 (0.64)	0.006
Frequency of PA with friends ^2^	2.44 (0.61)	2.38 (0.62)	2.49 (0.61)	<0.001
Frequency of extracurricular sports activity ^2^	2.25 (0.76)	2.28 (0.74)	2.23 (0.76)	0.159
Frequency of activity in a sports club ^2^	1.88 (0.91)	1.83 (0.91)	2.01 (0.92)	<0.001
Intensity of daily PA ^3^	2.25 (0.65)	2.18 (0.64)	2.34 (0.64)	<0.001
Playing football ^4^	2.12 (3.14)	6.44 (4.12)	2.64 (2.60)	0.588
Walking ^4^	3.43 (3.14)	3.97 (2.90)	4.67 (3.02)	0.782
Cycling ^4^	3.45 (2.71)	4.28 (2.63)	3.76 (2.38)	0.003
Running ^4^	3.62 (3.53)	5.26 (3.23)	5.25 (3.07)	0.424
Watching TV daily ^5^	3.91 (1.66)	3.73 (1.59)	4.05 (1.69)	<0.001
Using computer daily ^5^	3.10 (1.69)	2.68 (1.42)	3.55 (1.84)	<0.001
Playing digital games daily ^5^	2.97 (1.75)	2.42 (1.41)	3.55 (1.88)	<0.001

^1^ Measured on a 6-point scale: 1 = never to 6 = every day. ^2^ Measured on a 3-point scale: 1 = never to 3 = often. ^3^ Measured on a 3-point scale: 1 = light PA, 2 = moderate PA, and 3 = vigorous PA. ^4^ Measured on a 3-point scale: 1 = most often to 3 = almost never. ^5^ Measured on an 8-point scale: 1 = almost never to 8 = 6 or more hours per day. ^6^ *t*-test for independent samples.

**Table 4:** Academic competence in the total sample and differences between genders.

Academic Competence ^1^	Total Sample (*n* = 1520)	Females (*n* = 789)	Males (*n* = 731)	*p*-Value ^2^
	Mean (SD)	Mean (SD)	Mean (SD)	
Overall academic performance	3.80 (1.09)	3.98 (1.00)	3.64 (1.13)	<0.001
Reading performance	3.84 (1.15)	4.13 (0.99)	3.54 (1.23)	<0.001
Mathematics performance	3.75 (1.10)	3.82 (1.05)	3.70 (1.14)	0.034
Reading skills	3.86 (1.12)	4.12 (0.99)	3.58 (1.20)	<0.001
Mathematics skills	3.78 (1.08)	3.83 (1.04)	3.73 (1.12)	0.059
Overall motivation for school success	3.89 (1.06)	4.12 (0.94)	3.66 (1.12)	<0.001
Parental encouragement	4.06 (0.99)	4.20 (0.93)	3.93 (1.04)	<0.001
Intellectual functioning	3.87 (1.05)	3.99 (0.99)	3.75 (1.09)	<0.001
Overall classroom behavior	3.92 (1.04)	4.19 (0.90)	3.65 (1.11)	<0.001
Academic competence total score	34.76 (8.57)	36.39 (7.76)	33.17 (9.02)	<0.001

^1^ Measured using the SSRS test (teacher form) [36] on a 5-point scale based on the position of the student’s academic achievement in a class: 1 = the lowest 10%, 2 = the next-lowest 20%, 3 = the middle 40%, 4 = the next-highest 20%, and 5 = the highest 10% of students in the class. ^2^ *t*-test for independent samples.

**Table 5:** Three levels of academic competence of the total sample and female and male participants.

Level of Academic Competence ^1^	Total Sample (*n* = 1520)	Females (*n* = 789)	Males (*n* = 731)	*p*-Value ^2^
	*n* (%)	*n* (%)	*n* (%)	
Below average	216 (14.0)	68 (8.6)	141 (19.3)	<0.001
Average	1065 (68.8)	553 (70.1)	495 (67.7)	
Above average	266 (17.2)	168 (21.3)	95 (13.0)	

^1^ The total score of academic competence on the SSRS test was divided into three-level subgroups based on the calculation by Gresham and Elliott [36]. ^2^ Pearson’s chi-square test.

**Table 6:** Participants’ leisure-time PA by three subgroups of academic competence.

Leisure-Time PA	Level of Academic Competence			
	Below Average (*n* = 209)	Average (*n* = 1048)	Above Average (*n* = 263)	*p*-Value ^6^
	Mean (SD)	Mean (SD)	Mean (SD)	
Frequency of leisure-time PA ^1^	4.72 (1.53)	4.98 (1.19)	5.08 (0.97)	0.008
Frequency of PA performed alone ^2^	2.34 (0.64)	2.35 (0.62)	2.47 (0.55)	0.024
Frequency of PA in the family ^2^	2.09 (0.73)	2.17 (0.63)	2.24 (0.57)	0.054
Frequency of PA with friends ^2^	2.44 (0.66)	2.46 (0.60)	2.44 (0.58)	0.868
Frequency of extracurricular sports activity ^2^	2.23 (0.79)	2.24 (0.76)	2.31 (0.75)	0.438
Frequency of activity in a sports club ^2^	1.67 (0.85)	1.87 (0.92)	1.96 (0.93)	0.005
Intensity of daily PA ^3^	2.14 (0.71)	2.29 (0.65)	2.29 (0.61)	0.014
Playing football ^4^	1.87 (2.85)	2.02 (2.92)	2.35 (3.46)	0.222
Walking ^4^	2.73 (2.83)	3.55 (3.24)	3.69 (3.06)	0.003
Cycling ^4^	3.12 (3.06)	3.49 (2.68)	3.73 (2.64)	0.077
Running ^4^	3.55 (3.56)	3.62 (3.58)	4.11 (3.29)	0.158
Watching TV daily ^5^	4.01 (1.83)	3.99 (1.66)	3.73 (1.64)	0.108
Using computer daily ^5^	3.42 (1.79)	3.17 (1.73)	2.61 (1.51)	<0.001
Playing digital games daily ^5^	3.22 (1.92)	3.03 (1.74)	2.58 (1.53)	<0.001

^1^ Measured on a 6-point scale: 1 = never to 6 = every day. ^2^ Measured on a 3-point scale: 1 = never to 3 = often. ^3^ Measured on a 3-point scale: 1 = light PA, 2 = moderate PA, and 3 = vigorous PA. ^4^ Measured on a 3-point scale: 1 = most often to 3 = almost never. ^5^ Measured on an 8-point scale: 1 = almost never to 8 = 6 or more hours per day. ^6^ One-way ANOVA.

**Table 7:** Multiple regression models for predicting academic competencies in females and males.

Regression Models ^1^	Overall Academic Competence		Mathematical Competence ^3^		Reading Competence ^3^	
	*R* ^2^	*p*-Value	*R* ^2^	*p*-Value	*R* ^2^	*p*-Value
**Females**						
Model 1 (PA ^2^)	0.032	0.004	0.031	0.005	0.030	0.007
Model 2 (PA, grade level, developmental handicap)	0.171	<0.001	0.155	<0.001	0.123	<0.001
Model 3 (PA, grade level, developmental handicap, parental encouragement, family size)	0.552	<0.001	0.439	<0.001	0.423	<0.001
Model 4 (PA, grade level, developmental handicap, parental encouragement, family size, school type, level of teacher education and work experience)	0.572	0.022	0.435	0.208	0.524	0.055
Model 5 (PA, grade level, developmental handicap, parental encouragement, family size, school type, level of teacher education and work experience, students’ classroom behavior and motivation to succeed academically)	0.811	<0.001	0.552	0.084	0.779	<0.001
**Males**						
Model 1 (PA ^2^)	0.042	0.004	0.038	0.007	0.030	0.032
Model 2 (PA, grade level, developmental handicap)	0.319	<0.001	0.337	<0.001	0.261	<0.001
Model 3 (PA, grade level, developmental handicap, parental encouragement, family size)	0.542	<0.001	0.522	<0.001	0.406	<0.001
Model 4 (PA, grade level, developmental handicap, parental encouragement, family size, school type, level of teacher education and work experience)	0.799	<0.001	0.745	0.005	0.800	<0.001
Model 5 (PA, grade level, developmental handicap, parental encouragement, family size, school type, level of teacher education and work experience, students’ classroom behavior and motivation to succeed academically)	0.841	<0.001	0.819	0.002	0.835	<0.001

^1^ The calculations are based on the multiple regression analysis method Enter. ^2^ Predicted variables of leisure-time PA were selected as statistically significant variables in Table 6. ^3^ Mathematical and reading competencies were calculated as the mean score of academic performance and ability assessments.

**Table 8:** Associations between leisure-time PA and overall academic competence in females and males (Model 1).

Leisure-Time Physical Activity Predictors	Overall Academic Competence (Females)			Overall Academic Competence (Males)		
	*β* ^1^	95% Cl	*p*-Value	*β* ^1^	95% Cl	*p*-Value
Frequency of leisure-time PA	0.095	0.01 to 0.15	0.024	0.062	−0.03 to 0.15	0.199
Frequency of PA performed alone	0.002	−0.13 to 0.14	0.960	0.054	−0.07 to 0.26	0.240
Frequency of activity in a sports club	0.068	−0.02 to 0.17	0.105	0.150	0.07 to 0.30	0.002
Intensity of daily PA	0.067	−0.03 to 0.24	0.134	0.008	−0.17 to 0.19	0.877
Walking	−0.002	−0.03 to 0.03	0.970	−0.002	−0.04 to 0.03	0.960
Using computer daily	0.009	−0.05 to 0.06	0.832	−0.091	−0.12 to 0.01	0.089
Playing digital games daily	−0.076	−0.11 to 0.01	0.074	0.058	−0.03 to 0.10	0.280

^1^ Standardized regression coefficient (*β*), multiple regression analysis, and method Enter.

## Data Availability

The dataset related to this manuscript can be made available upon reasonable request.
